# Continuous overexpression of thioredoxin 1 enhances cancer development and does not extend maximum lifespan in male C57BL/6 mice

**DOI:** 10.1080/20010001.2018.1533754

**Published:** 2018-10-23

**Authors:** Lisa C. Flores, Madeline G. Roman, Geneva M. Cunningham, Christie Cheng, Sara Dube, Colton Allen, Holly Van Remmen, Gene B. Hubbard, Thomas L. Saunders, Yuji Ikeno

**Affiliations:** a Barshop Institute for Longevity and Aging Studies, The University of Texas Health Science Center at San Antonio, San Antonio, TX, USA; b Aging and Metabolism Research Program, Oklahoma Medical Research Foundation, Oklahoma City, OK, USA; c Department of Pathology, The University of Texas Health Science Center at San Antonio, San Antonio, TX, USA; d Transgenic Animal Model Core, University of Michigan, Ann Arbor, MI, USA; e Geriatric Research Education and Clinical Center (GRECC), Audie L. Murphy VA Hospital, South Texas Veterans Health Care System, San Antonio, TX, USA

**Keywords:** Thioredoxin (Trx), transgenic mouse (Tg), oxidative stress, aging, cancer

## Abstract

We examined the effects of continuous overexpression of thioredoxin (Trx) 1 on aging in Trx1 transgenic mice [Tg(*TXN*)^+/0^]. This study was conducted to test whether increased thioredoxin expression over the lifespan in mice would alter aging and age-related pathology because our previous study demonstrated that Tg(act-*TXN*)^+/0^ mice had no significant maximum life extension, possibly due to the use of actin as a promoter, which may have resulted in loss of Trx1 overexpression during aging. To test this hypothesis, we generated new Trx1 transgenic mice using a fragment of the human genome containing the *TXN* gene with an endogenous promoter to ensure continuous overexpression of Trx1 throughout the lifespan. Universal overexpression of Trx1 was observed, and Trx1 overexpression was maintained during aging (up to 22–24 months old) in the Tg(*TXN*)^+/0^ mice. The levels of Trx1 are significantly higher (approximately 4 to 31 fold) in all of the tissues examined in the Tg(*TXN*)^+/0^ mice compared to the wild-type (WT) littermates. The overexpression of Trx1 did not cause any changes in the levels of Trx2, glutaredoxin, glutathione, or other major antioxidant enzymes. The survival study demonstrated that male Tg(*TXN*)^+/0^ mice slightly extended the earlier part of the lifespan compared to WT littermates, but no significant life extension was observed over the lifespan. The cross-sectional pathological analysis (22–25 months old) showed that Tg(*TXN*)^+/0^ mice had a significantly higher severity of lymphoma and more tumor burden than WT mice, which was associated with the suppression of the apoptosis signal-regulating kinase 1 (ASK1) pathway. Our findings suggest that the increased levels of Trx1 over the lifespan in Tg(*TXN*)^+/0^ mice showed some beneficial effects (slight extension of lifespan) in the earlier part of life but had no significant effects on median or maximum lifespans, and increased Trx1 levels enhanced tumor development in old mice.

## Introduction

Thioredoxin (Trx) was first recognized in the early 1960s as the reductant for a variety of enzymes and has been found in various mammalian species. There are two forms of Trx in humans: 1) Trx1, which is located in the cytosol [1]; and 2) Trx2, which is located in the mitochondria [2]. Both Trx1 and 2 play essential biological roles in mammals. Some of the physiologically important roles of Trx include: 1) acting as a hydrogen donor for enzymes involved in reductive reactions [3–8]; 2) maintaining a reduced environment in cells [9]; 3) controlling protein (especially transcription factors) function via the redox state [10–12] and the expression of target genes; 4) protecting cells and tissues from oxidative stress [13]; and 5) having anti-apoptotic effects through the inhibition of the ASK [14] and mitochondrial pathways [15]. The essential biological roles of Trx were further supported by two studies which demonstrated that mice null for either Trx1 or Trx2 were embryonically lethal [16,17].

The important roles of Trx in oxidative stress and longevity have been well documented by studies with *C. elegans* and *Drosophila* [18,19]; however, the exact roles of Trx in aging and age-related diseases in mammals have not been fully explored. Thus, we previously conducted an aging study with Trx transgenic mice [Tg(act-*TXN*)^+/0^] [20] and demonstrated that Trx overexpression showed resistance to oxidative stress, which was associated with an extension of only the earlier part of lifespan compared to WT littermates [20]. Interestingly, the Tg(act-*TXN*)^+/0^ mice showed reduced levels of Trx1 overexpression with age and a slightly higher incidence of cancer compared to WT mice [20]. Therefore, we hypothesized that the Tg(act-*TXN*)^+/0^ mice did not show an increase in maximum lifespan because: 1) the overexpression of Trx1 in the Tg(act-*TXN*)^+/0^ mice is significantly reduced with age; and/or 2) Trx1 could promote cancer development in old age.

To test this hypothesis, we generated new transgenic mice with clones of the human *TXN* gene containing endogenous promoters to ensure that the transgene is overexpressed throughout the lifespan, and conducted a survival study. The purpose of this study, therefore, was to determine whether continuous overexpression of Trx1 over the lifespan could extend maximum lifespan and/or alter age-related pathology, especially cancer. Here, we report that the increased levels of Trx1 over the lifespan in the Tg(*TXN*)^+/0^ mice resulted in a slight extension of lifespan in the earlier part of life but showed no significant effects on maximum lifespan. The cross-sectional pathology showed that the old (22–25 months) Tg(*TXN*)^+/0^ mice had a significantly higher severity of lymphoma and more tumor burden than WT mice, which was correlated with the suppression of the ASK1 pathway. Our findings suggested that the increased levels of Trx1 over the lifespan in the Tg(*TXN*)^+/0^ mice showed no significant beneficial effects on maximum lifespan, possibly due to enhanced tumor development in old mice, which is consistent with our previous report [20].

## Materials and methods

### Animals and animal husbandry

**Figure 1.**
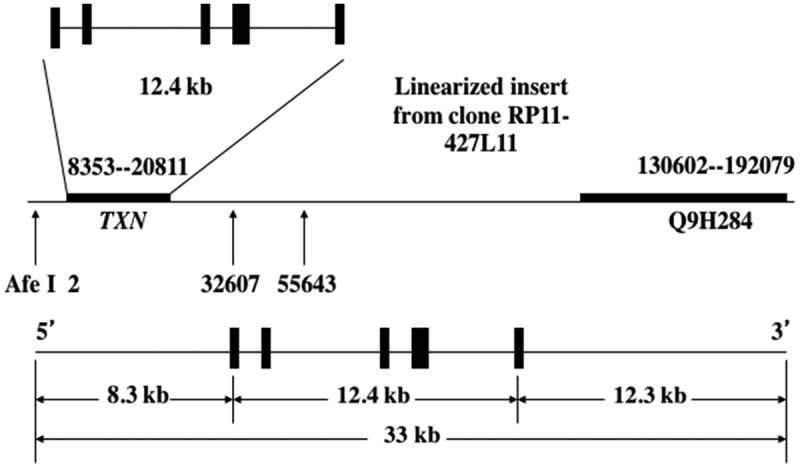
**DNA construct for the Tg(*TXN*)^+/0^ mice.** The human thioredoxin gene (*TXN)* with 8.3 kb and 12.3 kb of the 5ʹ- and 3ʹ-flanking sequences was used to generate Tg(*TXN*)^+/0^ mice by pronuclear microinjection of zygotes from the mating of (C57BL/6J X SJL/J)F1 females with (C57BL/6J X SJL/J)F1 males.

The Trx1 transgenic mice used in this study were generated using a fragment of the human genome containing the *TXN* gene [a BAC clone (RP11-427L11), Children’s Hospital Oakland Research Institute’s (CHORI) BACPAC Resources Center (BPRC), Oakland, CA] with 8.3 kb and 12.3 kb of the 5ʹ- and 3ʹ-flanking sequences, respectively (Figure 1). These transgenic mice were produced by pronuclear microinjection of zygotes obtained from the mating of (C57BL/6J X SJL/J)F1 females with (C57BL/6J X SJL/J)F1 males (Jackson Laboratory stock no. 100,012). They have been backcrossed to C57BL/6 mice ten times. Male hemizygous transgenic mice [Tg(*TXN*)^+/0^] were crossed with C57BL/6 females to generate hemizygous transgenic and WT control mice. All mice were fed a commercial chow (Teklad Diet LM485: Madison, WI) and acidified (pH = 2.6–2.7) filtered reverse osmosis water *ad libitum*. To measure the amount of food consumption, the amount of chow removed from the cage hopper and the spillage (the chow on the bottom of the cage) were weighed monthly. Actual food consumed was calculated by subtracting the spillage from the chow removed from the hopper. All of the mice were weighed monthly. The mice were maintained pathogen-free in microisolater units on Tek FRESH® ultra laboratory bedding. Sentinel mice housed in the same room and exposed weekly to bedding collected from the cages of experimental mice were sacrificed on receipt and every six months thereafter for monitoring of viral antibodies (Mouse Level II Complete Antibody Profile CARB, Ectro, EDIM, GDVII, LCM, M. Ad-FL, M. Ad-K87, MCMV, MHV, *M. pul*., MPV, MVM, Polyoma, PVM, Reo, Sendai; BioReliance, Rockville, MD). All tests were negative.

We chose young (3–7 months) and old (22–25 months) age groups for the experiments described below because: 1) C57BL/6 mice reach their optimum reproductivity at 3–7 months of age; and 2) have increased incidence of cancers after 20 months of age followed by advanced stages of cancer around 26–30 months of age. For some of the assays, only liver was used because there is a large amount of information available with respect to age-related changes in liver, and liver is one of the major sites for the development of spontaneous tumors in C57BL/6 mice.

### Determination of Trx1 expression

Cytosolic fractions obtained from tissues homogenized as previously described [20] were used to determine Trx1 levels in several tissues (liver, brain, heart, kidney, spleen, and lung) obtained from young (3 to 4 month old) and old (22 to 24 month old) Tg(*TXN*)^+/0^ and WT mice by Western blot analysis using goat anti-human Trx1 polyclonal antibodies (Catalog No. 705; American Diagnostica, Inc., Greenwich, CT). These antibodies recognize total Trx1 (both oxidized and reduced forms). After incubation with the primary antibody, membranes were incubated with the peroxidase-linked secondary antibody (Catalog No. P0449; Dako, Carpinteria, CA). Chemiluminescence was detected with an ECL Western blot detection kit (Amersham Biosciences Corp., Piscataway, NJ).

### Thioredoxin 2, glutaredoxin, peroxiredoxin, and total gluthathione levels

Thioredoxin 2 (Trx2), glutaredoxin (Grx), and peroxiredoxin 1 and 2 levels were measured using the mitochondrial (for Trx2 and peroxiredoxin 2) and total homogenate fractions (for glutaredoxin and peroxiredoxin 1) obtained from the livers of young (6 to 7 month) and old (22 to 24 month) Tg(*TXN*)^+/0^ and WT mice as previously described [20–22]. Western blot analysis was performed using goat anti-human glutaredoxin polyclonal antibody (Catalog No. 710; American Diagnostica, Inc., Greenwich, CT), rabbit anti-Trx2 polyclonal antibody (Catalog No. LF-PA0012; LabFrontier, Seoul, Korea), rabbit anti-human peroxiredoxin 1 polyclonal antibody (Catalog No. 210–521-R100; Alexis Biochemicals, San Diego, CA), and rabbit anti-human peroxiredoxin 2 polyclonal antibody (Catalog No. 210–522-R100; Qbiogene Inc., Alexis Biochemicals, Carlsbad, CA). After incubation with the primary antibodies, membranes were incubated with the respective peroxidase-linked secondary antibodies (Catalog Nos. P0449 and P0217; Dako, Carpinteria, CA). Chemiluminescence was detected using the ECL Western blot detection kit (Amersham Biosciences Corp., Piscataway, NJ). The levels of total glutathione were determined using the Bioxytech GSH-420 kit (Catalog No. 21,023; Oxis International, Inc., Foster City, CA).

### Determination of major antioxidant enzyme activities: Cu/ZnSOD, MnSOD, glutathione peroxidase, and catalase

The activities of major antioxidant enzymes (Cu/ZnSOD, MnSOD, glutathione peroxidase (GPx), and catalase) were measured in tissue homogenates obtained from the livers of young (3 to 4 month) Tg(*TXN*)^+/0^ and WT mice. The supernatants were used for the antioxidant defense enzymatic activity assay. GPx activity in tissue homogenates was measured by the assay as described by Sun *et al*. [23]. Catalase activity was determined by measuring the decomposition of hydrogen peroxide at 520 nm using the Catalase-520^TM^ assay kit (*Oxis*Research^TM^, Portland, OR). MnSOD and Cu/ZnSOD levels were measured by activity gels as previously described [24]. Images of the gels were analyzed by ImageQuant software.

### Assays for lipid peroxidation (F2-isoprostane levels)

The levels of F2-isoprostanes were determined using gas chromatography/mass spectrometry as described by Morrow and Roberts [25]. The plasma samples obtained from 23 month old mice were added to HPLC (pH 3.0) water and mixed by vortex. After centrifugation (2,500 x *g* for 3 minutes at 4°C), the F2-isoprostanes were extracted from the clear supernatants with a C18 Sep-Pak column and a silica Sep-Pak column. The F2-isoprostanes were then converted to pentafluorobenzyl esters and subjected to thin layer chromatography. The F2-isoprostanes were further converted to trimethylsilyl ether derivatives, and the F2-isoprostane levels were quantified by gas chromatography/mass spectrometry. An internal standard, 8-isoPGF2a-d4 (Cayman Chemical, Ann Arbor, MI), was added to the samples at the beginning of extraction to correct the yield of the extraction process. The amounts of F2-isoprostanes were expressed as nanograms of 8-Iso-prostaglandin F2 per milliliter of plasma sample.

### Determination of ASK1 signaling pathway activity

The levels of ASK1 were measured by Western blot using an ASK1 antibody (Santa Cruz, CA). The activation of ASK1 was also measured by Western blot with a phospho-ASK1 antibody [Thr845] (Cell Signaling Technology, Inc., MA). The liver obtained from young (6–7 months old) and old (22–24 months old) Tg(*TXN*)^+/0^ and WT mice were homogenized in lysis buffer (20 mM Tris-HCl [pH 7.5], 150 mM NaCl, 1 mM EDTA, 10% glycerol, 1% NP-40, and 1X protease inhibitor cocktail I [Calbiochem]) and incubated on ice for 30 minutes. After centrifugation, the supernatant was separated, and the protein concentration was determined by the Bradford assay. Proteins (100 μg) were separated on polyacrylamide gel electrophoresis (PAGE) and transferred onto nitrocellulose membrane electrophoretically. Specific proteins on the membranes were detected by standard Western blotting procedures using secondary antibodies conjugated to horseradish peroxidase (HRP). Signals were detected with an ECL Western blot detection kit (Amersham Biosciences Corp., Piscataway, NJ).

### Measurement of the mitochondrial apoptosis pathway

The mitochondrial apoptosis pathway was assessed by measuring caspase activity, cytochrome c release from the mitochondria, and bcl-2 levels in the liver obtained from young (6–7 months old) Tg(*TXN*)^+/0^ and WT mice. The caspase activities, cytochrome c release, and bcl-2 levels were determined by Western blot assay. The caspase-3 activity in liver tissue were visualized by the cleavage forms of caspase-3 (p20) as an indicator of caspase activity using anti-caspase-3 antibody (Cell Signaling Technology, Inc., Danvers, MA). The intensities of cleavage bands corresponding to p20 for caspase-3 were quantified by densitometry using ImageQuant v5.0, and β-actin was used as a loading control. The cytochrome c release from mitochondria was measured as levels of cytochrome c in the cytosolic fractions from liver. Equal amounts of protein were separated on a 4–20% SDS-polyacrylamide gel, transferred to nitrocellulose membranes, and subjected to Western blotting with an anti-cytochrome c antibody (Santa Cruz Biotechnology, Santa Cruz, CA). The bcl-2 levels were also measured by Western blot using anti-bcl-2 antibody (Cell Signaling Technology, Inc., Danvers, MA). Data were normalized to β-actin loading controls. The data were expressed as relative units respective to β-actin.

### Survival study

Mice in the survival groups were allowed to live out their lives, and the lifespan for individual mice was determined by recording the age of spontaneous death. A survival study consisting of 47 male Tg(*TXN*)^+/0^ and 58 male WT mice was conducted. The survival curves were compared statistically using the log-rank test [26]. The median, mean, 10^th^ percentile (when 90% of the mice had died), and maximum survivals were calculated for each group. The mean survivals for each experimental group were compared to the respective WT group by performing a Student’s t-test upon log-transformed survival times. The median and 90^th^ percentile survivals for each group were compared to the WT group using a score test adapted from Wang *et al*. [27].

### Cross–sectional pathological assessment

After the gross pathological examinations, the following organs and tissues were excised and preserved in 10% buffered formalin: brain, pituitary gland, heart, lung, trachea, thymus, aorta, esophagus, stomach, small intestine, colon, liver, pancreas, spleen, kidneys, urinary bladder, reproductive system (prostate, testes, epididymis, and seminal vesicles), thyroid gland, adrenal glands, parathyroid glands, psoas muscle, knee joint, sternum, and vertebrae. Any other tissues with gross lesions were also excised. The fixed tissues were processed conventionally, embedded in paraffin, sectioned at 5 μm, and stained with hematoxylin-eosin. The diagnosis of each histopathological change was made with histological classifications in aging mice as previously described [28,29]. A list of pathological lesions was constructed for each mouse that included both neoplastic and non-neoplastic diseases. Based on these histopathological data, the tumor burden, disease burden, and severity of each lesion in each mouse were assessed as previously described [20,29–31]. The cross-sectional pathological analyses were conducted with 38 Tg(*TXN*)^+/0^ mice and 48 WT male mice.

### Statistical analysis

Unless otherwise specified, all experiments were done at least in triplicate. Data were expressed as means ± SEM and were analyzed by the non-parametric test ANOVA. All pair-wise contrasts were computed using Tukey error protection at 95% CI, unless otherwise indicated. Differences were considered statistically significant at *p *< 0.05.

## Results

### Overexpression of Trx1 in tissues from Tg(TXN)^+/0^ mice

**Figure 2.**
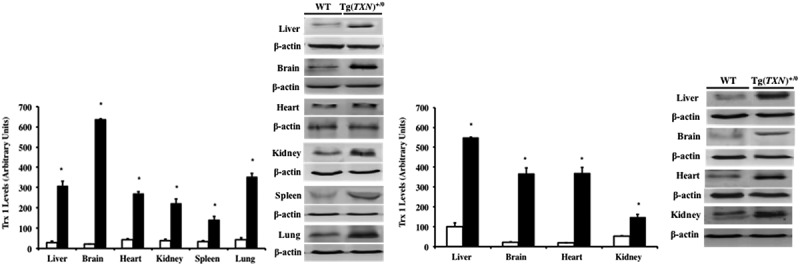
**The overexpression of Trx1 in young and old age Tg(*TXN*)^+/0^ mice and their WT littermates.** The levels of Trx1 protein were determined by Western blot in various tissues of 3 to 4 month old Tg(*TXN*)^+/0^ (closed bars) and WT mice (open bars) (Figure 2(a): left). The data are the mean ± SEM from three animals. Trx1 was significantly higher in the livers of Tg(*TXN*)^+/0^ mice compared to their WT littermates (*p < 0.05). Trx1 was also significantly higher in the tissues of 22 to 24 month old Tg(*TXN*)^+/0^ (closed bars) and WT mice (open bars) (Figure 2(b): right) (*p < 0.05). The data are the mean ± SEM from three to five mice.

The levels of Trx1 in tissues from young (3–4 months old; liver, brain, heart, kidney, spleen, and lung) and old (22–24 months old; liver, brain, heart, and kidney) Tg(*TXN*)^+/0^ and WT mice were measured using Western blot analysis. The Trx1 protein levels were significantly higher (approximately 4 to 31 fold) in all of the six tissues examined in the young Tg(*TXN*)^+/0^ mice compared to their WT littermates (Figure 2(a)). The levels of Trx1 overexpression were maintained in old aged mice (Figure 2(b)).

### Survival curves, body and organ weights

**Figure 3.**
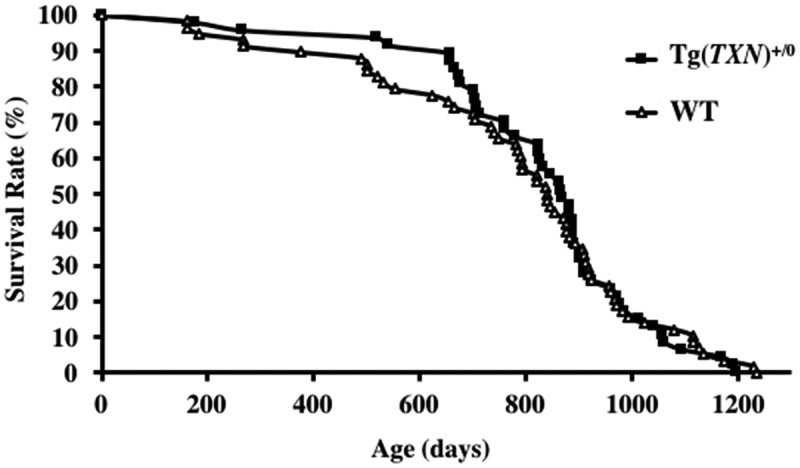
**The survival curves of Tg(*TXN*)^+/0^ and WT mice.** The survival curves of Tg(*TXN*)^+/0^ (closed squares) and WT (open triangles) mice are presented. The cohort consists of 47 Tg(*TXN*)^+/0^ mice and 58 WT male mice. The survival curves did not show a significant difference between Tg(*TXN*)^+/0^ and WT mice (*p *> 0.05).

The survival curves of Tg(*TXN*)^+/0^ and WT mice are presented in Figure 3. The survival study was conducted with 47 male Tg(*TXN*)^+/0^ and 58 male WT mice. The body and organ weights were similar between Tg(*TXN*)^+/0^ and WT mice (Table 1). Food consumption was also similar between Tg(*TXN*)^+/0^ and WT mice (data not shown). The survival curves were not significantly different between Tg(*TXN*)^+/0^ and WT mice. The male Tg(*TXN*)^+/0^ mice showed slight increases in earlier survival; however, we found no significant life extension (mean, median, and 10^th^ percentile survival) over the lifespan.

**Table 1.** Body and organ weights of Tg(*TXN*)^+/0^ mice.

	WT (n = 5)	Tg(*TXN*)^+/0^ (n = 8)
Body Weight (g)	27.286 ± 0.470	26.972 ± 0.525
Liver (g)	1.165 ± 0.030	1.219 ± 0.044
Spleen (g)	0.085 ± 0.002	0.088 ± 0.006
Pancreas (g)	0.147 ± 0.002	0.150 ± 0.011
Heart (g)	0.158 ± 0.005	0.150 ± 0.004
Lung (g)	0.173 ± 0.007	0.185 ± 0.008
Left Kidney (g)	0.231 ± 0.006	0.215 ± 0.008
Right Kidney (g)	0.250 ± 0.015	0.233 ± 0.010
Left Testicle (g)	0.073 ± 0.021	0.097 ± 0.007
Right Testicle (g)	0.080 ± 0.021	0.107 ± 0.004
Brain (g)	0.510 ± 0.054	0.437 ± 0.009

### Cross-sectional pathology

**Figure 4.**
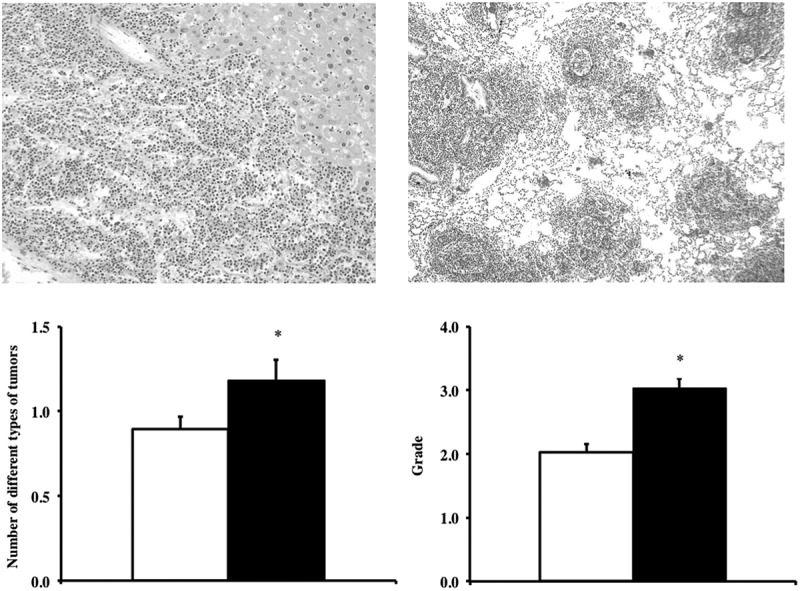
**Tumor burden and severity of lymphoma in Tg(*TXN*)^+/0^ and WT mice.** Photographic images of liver (Figure 4(a): left, top, H&E 200x) and lung (Figure 4(b): right, top, H&E 100x) show high-grade lymphoma development in these tissues in Tg(*TXN*)^+/0^ mice. The cohort consists of 38 Tg(*TXN*)^+/0^ mice and 48 WT male mice. The number of different types of tumors, tumor burden (Figure 4(c): left, bottom) and the severity of lymphoma (Figure 4(d): right, bottom) in Tg(*TXN*)^+/0^ (closed bar) and WT (open bar) mice were compared (22–25 months old). The tumor burden for the Tg(*TXN*)^+/0^ mice is significantly higher (24.4% higher) than that of WT mice (*p = 0.043), which suggests that the overall tumor incidence is higher in the Tg(*TXN*)^+/0^ mice compared to WT control mice. The severity of lymphoma was significantly higher in Tg(*TXN*)^+/0^ mice compared to their WT littermates (*p = 0.0001).

The cross-sectional pathological analyses of 38 Tg(*TXN*)^+/0^ and 48 WT mice (22–25 months) showed that the major disease in these mice was neoplastic disease, especially lymphoma (Figure 4(a,b)), which is consistent with the end-of-life pathology data from C57BL/6 mice and Tg(act-*TXN*)^+/0^ mice [20,28,29]. The tumors originated in liver (Figure 4(a)), spleen and mesenteric lymph nodes, and tumor cells were also involved in kidney, lung (Figure 4(b)), and other tissues as severity increased. Other tumors observed were hemangioma/hemangiosarcoma in the liver and spleen, pulmonary adenocarcinoma, hepatocellular carcinoma, and adenoma in the thyroid gland.

First, we compared the number of different types of tumors (tumor burden) for each mouse in both Tg(*TXN*)^+/0^ and WT groups because aging mice may have several different types of tumors. As the data in Figure 4(c) show, the tumor burden for the Tg(*TXN*)^+/0^ mice is significantly higher (24.4% higher) than that of WT mice (*p *= 0.043), suggesting that the overall tumor incidence is higher in the Tg(*TXN*)^+/0^ mice than WT mice, which is similar to our previous study that showed the incidence of fatal tumors was higher in the Tg(act-*TXN*)^+/0^ compared to WT mice [20]. The incidence of lymphoma was similar between Tg(*TXN*)^+/0^ (89.5%) and WT mice (81.3%). However, the Tg(*TXN*)^+/0^ mice had a significantly higher severity of lymphoma than WT mice (*p *= 0.0001; Figure 4(d)).

**Figure 5.**
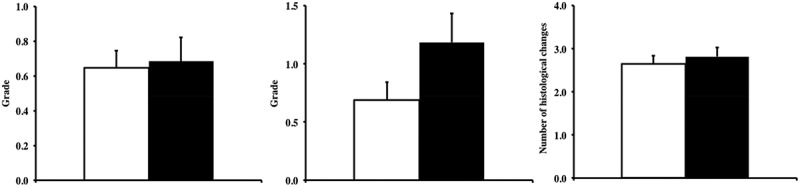
**Glomerulonephritis, inflammation, and disease burden in Tg(*TXN*)^+/0^ and WT mice.** The severity of glomerulonephritis (Figure 5(a): left), and inflammation (Figure 5(b): center), and total number of pathological changes (disease burden: Figure 5(c): right) were determined in Tg(*TXN*)^+/0^ (closed bar) and WT (open bar) mice (22–25 months old). The cohort consists of 38 Tg(*TXN*)^+/0^ mice and 48 WT male mice. The disease burden in Tg(*TXN*)^+/0^ mice was similar to WT mice. No significant changes were found in the severity of glomerulonephritis or inflammation in Tg(*TXN*)^+/0^ mice compared to WT mice.

Next, we compared the severity of major non-neoplastic disease between the two groups. Glomerulonephritis and inflammation were the most common non-neoplastic lesions observed in these mice, and we compared the severity of these lesions. No significant changes were found regarding the severity of glomerulonephritis or inflammation (Figure 5(a,b); *p *> 0.05), although Tg(*TXN*)^+/0^ mice had a slightly higher severity of inflammation compared to WT mice.

The disease burden, defined as the total number of histopathological changes in a body, can serve as a good index of age-related accumulation of tissue and cell injury [31], and more importantly, less disease burden was correlated to extended lifespans in long-lived animal models [30,31]. Figure 5(c) shows a comparison of the disease burden between the Tg(*TXN*)^+/0^ and WT mice, indicating that the Tg(*TXN*)^+/0^ mice had a similar (*p *> 0.05) disease burden compared to WT mice.

Therefore, the continuous overexpression of Trx1 plays more important roles in tumor development, especially lymphoma than other age-related diseases in Tg(*TXN*)^+/0^ mice.

### Lipid peroxidation (F2-isoprostane levels)

**Figure 6.**
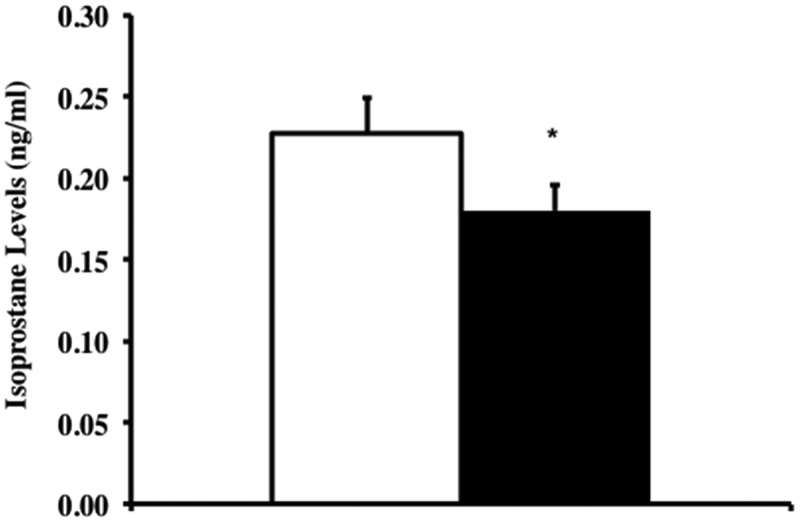
**Levels of F_2_-isoprostanes in Tg(*TXN*)^+/0^ and WT mice.** Plasma samples from Tg(*TXN*)^+/0^ (closed bar) and WT (open bar) mice were processed, and F_2_-isoprostanes levels were measured in 23 month old mice. The F_2_-isoprostane levels in plasma of Tg(*TXN*)^+/0^ mice were significantly lower than in WT control mice (*p < 0.05). The data are the mean ± SEM from five mice.

To test whether Trx1 overexpression reduced oxidative damage and its effects on aging in the Tg(*TXN*)^+/0^ mice compared to WT mice, we measured the levels of lipid peroxidation (F_2_ isoprostanes) in plasma from old (23 months) Tg(*TXN*)^+/0^ and WT mice. Levels of F_2_-isoprostanes in plasma were significantly lower (22% less, Figure 6) in the Tg(*TXN*)^+/0^ mice compared to WT mice, which could indicate that whole-body oxidative stress is lower in the Tg(*TXN*)^+/0^ mice compared to WT mice.

### ASK1 levels

**Figure 7.**
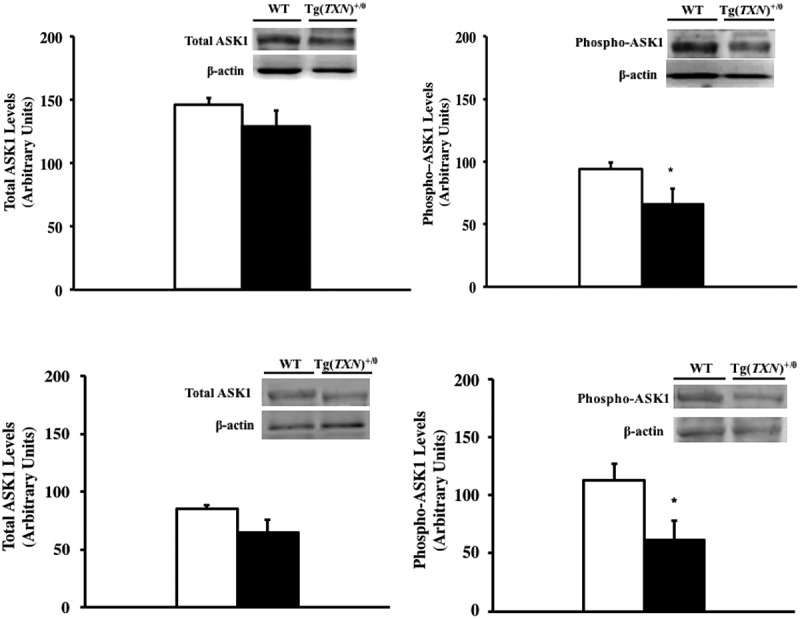
**Levels of total ASK1 and phosphorylated ASK1 in Tg(*TXN*)^+/0^ and WT mice.** The levels of ASK1 and phosphorylated ASK1 were measured in the livers of young (6–7 months old: Figure 7(a,b)) and old (22–24 months old: Figure 7(c,d)) Tg(*TXN*)^+/0^ (closed bar) and WT (open bar) mice by Western blot analysis. The levels of ASK1 (Figure 7(a,c)) were similar between Tg(*TXN*)^+/0^ and WT mice. However, phosphorylated ASK1 levels (Figure 7(b,d)) were significantly lower in young and old Tg(*TXN*)^+/0^ compared to WT mice (*p < 0.05). The data are the mean ± SEM from three to five mice.

As we have previously shown that Tg(act-*TXN*)^+/0^ mice had higher levels of the ASK1/Trx1 complex, which could result in reduced ASK1 phosphorylation [20] that was correlated to tumor development, we measured the levels of ASK1 and phosphorylated ASK1 young (6–7 months old: Figure 7(a,b)) and old (22–24 months old: Figure 7(c,d)) Tg(*TXN*)^+/0^ and WT mice.

Figure 7(a,c) show levels of ASK1 were similar between Tg(*TXN*)^+/0^ mice compared to WT mice (both young and old groups). However, phosphorylated ASK1 levels were significantly lower in Tg(*TXN*)^+/0^ mice compared to WT mice (both young and old groups: Figure 7(b,d)).

### Measurement of mitochondrial apoptosis pathway

**Figure 8.**
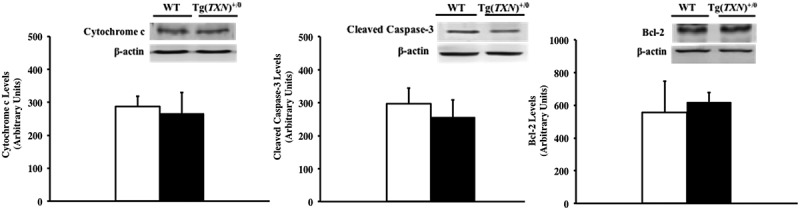
**Cytochrome c release, caspase-3 activity, and bcl-2 levels in Tg(*TXN*)^+/0^ and WT mice.** Cytochrome c release (Figure 8(a): left), caspase-3 activity (Figure 8(b): center), and bcl-2 levels (Figure 8(c): right) were measured by Western blot analysis to assess the mitochondrial apoptosis pathway in the livers of 6 to 7 month old Tg(*TXN*)^+/0^ (closed bar) and WT (open bar) mice. Cytochrome c release, caspase-3 activity, and bcl-2 levels were similar between Tg(*TXN*)^+/0^ and WT mice. The values are the mean ± SEM of five mice per group.

The mitochondrial apoptosis pathway was also assessed by caspase activity, cytochrome c release from the mitochondria, and bcl-2 levels because Trx1 overexpression altered the ASK1 pathway. Cytochrome c release, caspase-3 activity, and bcl-2 levels were similar between young (6–7 months old) Tg(*TXN*)^+/0^ and WT mice (Figure 8(a–c)).

### Levels of Trx2, glutaredoxin, glutathione, and peroxiredoxin 1 and 2 in tissues from Tg(TXN)^+/0^ mice

**Figure 9.**
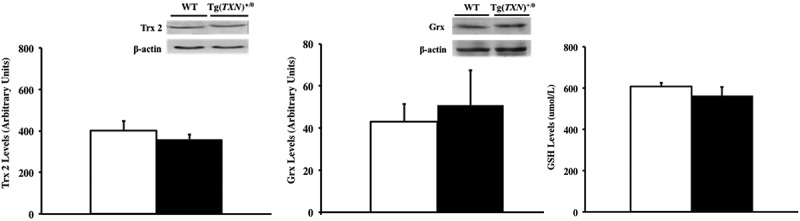
**Levels of Trx2, glutaredoxin, and total glutathione in Tg(*TXN*)^+/0^ and WT mice.** The levels of Trx2 (Figure 9(a): left), glutaredoxin (Figure 9(b): center), and total glutathione (Figure 9(c): right) were measured in the livers of young 6 to 7 month Tg(*TXN*)^+/0^ mice (closed bar) and WT mice (open bar) by Western blot. No significant difference was observed in Trx2, glutaredoxin, or total glutathione in Tg(*TXN*)^+/0^ mice compared to WT mice. The data in Figure 9(a–c) are the mean ± SEM from three to five mice.

We also determined whether the levels of Trx2, glutaredoxin, and glutathione were altered in response to increased Trx levels because biological functions of Trx2 (mitochondrial form of Trx), glutathione, and glutaredoxin complement Trx1. The data in Figure 9 show that no significant changes in Trx2 (Figure 9(a)) and glutaredoxin (Figure 9(b)) levels were observed in the livers of young (6–7 months old) Tg(*TXN*)^+/0^ and WT control mice. The Tg(*TXN*)^+/0^ and WT control mice showed similar glutathione levels in the liver (Figure 9(c)) at 6–7 months of age. The levels of Trx2, glutaredoxin, and glutathione were also similar between old (22–24 months old) Tg(*TXN*)^+/0^ and WT mice (data not shown).

**Figure 10.**
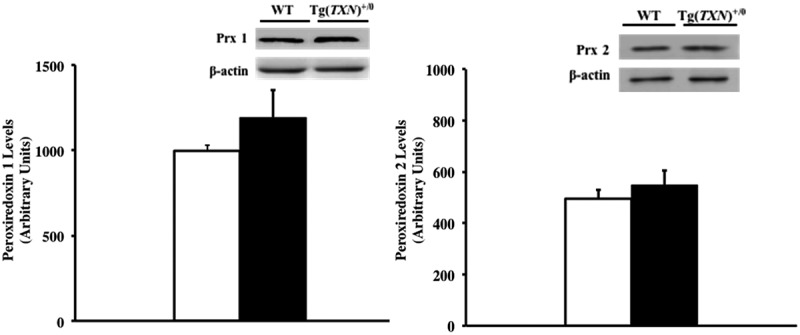
**Levels of Prx 1 and Prx 2 in Tg(*TXN*)^+/0^ and WT mice.** The levels of Prx 1 (Figure 10(a): left) and Prx 2 (Figure 10(b): right) were measured in the livers from young (6 to 7 months old) Tg(*TXN*)^+/0^ mice (closed bar) and WT control mice (open bar). No significant differences were observed in Prx 1 or Prx 2 levels of Tg(*TXN*)^+/0^ mice compared to WT mice. The data in the figures are the mean ± SEM from three to five mice.

In addition, levels of peroxiredoxin (Prx) 1 and 2 in the livers of young (6–7 months old) Tg(*TXN*)^+/0^ and WT control mice were measured because one of the major roles of Trx is as a hydrogen donor for Prxs in reductive reactions, which reduce peroxides [3,4]. The data in Figure 10 show that no significant changes in Prx1 (Figure 10(a)) and Prx2 (Figure 10(b)) levels were observed in the livers of young (6–7 months old) Tg(*TXN*)^+/0^ and WT control mice.

Therefore, the data in Figures 9 and 10 show that Trx2, glutaredoxin, total glutathione, and Prx 1 & 2 levels were not altered by the overexpression of Trx1 in the Tg(*TXN*)^+/0^ mice.

### Major antioxidant enzyme activities in tissues from Tg(TXN)^+/0^ mice

The activities of the other major antioxidant enzymes were also measured in the liver obtained from young (3–4 months old) Tg(*TXN*)^+/0^ and WT mice to determine if an increase in Trx activity could initiate a compensatory reduction in the activities of other components of the antioxidant system. The activities of Cu/ZnSOD, MnSOD, catalase, and GPx were similar in the tissues from the Tg(*TXN*)^+/0^ and WT mice (data not shown). The data show that the major antioxidant enzyme activities were not altered by Trx1 overexpression in the tissues of the Tg(*TXN*)^+/0^ mice.

Thus, the overexpression of Trx1 plays more important roles in neoplastic diseases in old mice, i.e., increased the occurrence of neoplastic diseases and accelerated the development/growth of lymphoma compared to WT mice, caused in part by reduced apoptosis through the ASK1 pathway.

## Discussion

Thioredoxin (Trx) is a small protein (12kDa) with two redox-active cysteine residues in the active center (Cys-Gly-Pro-Cys), and was first recognized in the early 1960s as the reductant for a variety of enzymes [9]. It was shown that Trx was reduced by Trx reductase in a NADPH-dependent reaction and that in its dithiol form, Trx served as the reductant for methionine sulfoxide [6–8] and PAPS (3ʹ-phosphoadenosine-5ʹ-phosphosulfate) in yeast and of ribonucleotides in *Escherichia coli* [32,33]. Isoforms of Trx have been found in *E. coli*, yeast, and mammals. Two Trxs have been identified in humans, one cytosolic (hTrx1) [1] and one mitochondrial (hTrx2) [2]. Knockout mice null for either Trx1 or Trx2 are embryonically lethal [16,17], which demonstrates the essential roles of Trx1 and Trx2 in mammals. Trx1 plays an important role in maintaining the reduced environment in cells through thiol-disulfide exchange reactions. A major role of Trx is as a hydrogen donor for enzymes involved in reductive reactions, e.g., ribonucleotide reductase, which reduces ribonucleotides to deoxyribonucleotides for DNA synthesis; peroxiredoxins (Prxs) which reduce peroxides [3,4]; and methionine sulfoxide reductases, which reduce methionine sulfoxide in proteins and provide protection against oxidative stress [6–8]. All Trxs catalyze the reduction of disulfides in proteins more efficiently (orders of magnitude faster) than GSH or dithiothreitol [9]. Furthermore, Trx protects cells and tissues from oxidative stress [13,20].

In addition to its antioxidant property, Trx1, unlike other antioxidant enzymes, plays major roles in the maintenance of the cellular redox state and in redox signaling regulation [34]. Several studies have indicated that a disturbance in the redox state of an organism leads to aging and age-related diseases [35–37]. Due to these unique features, Trx, along with thioredoxin interacting protein (Txnip), has been shown to play very important roles in physiology and its dysregulation could be involved in various diseases [38]. However, the roles of Trx on aging and age-related pathology in mammals have not fully been investigated.

Therefore, we previously conducted an aging study to examine the effects of increased levels of Trx1 on longevity and its long-term pathophysiological consequences under optimal housing conditions, using a unique animal model overexpressing Trx1 [Tg(act-*TXN*)^+/0^ mice]. Although Tg(act-*TXN*)^+/0^ mice extended the earlier part of lifespan, no significant life-extension was observed in maximum lifespan. The loss of life-extending effects in older aged mice was associated with reduced Trx1 overexpression, possibly due to the use of actin as a promoter. In addition, the Tg(act-*TXN*)^+/0^ mice showed a slightly higher incidence of cancer, especially lymphoma, compared to WT mice. Based on these observations, we hypothesized that the Tg(act-*TXN*)^+/0^ mice did not show an increase in maximum survival for two reasons: 1) the overexpression of Trx1 in Tg(act-*TXN*)^+/0^ mice is significantly reduced with age; and 2) Trx1 could promote cancer development in old age. To test this hypothesis, we conducted a survival study using new transgenic mice we generated with clones of the human *TXN* gene containing endogenous promoters [Tg(*TXN*)^+/0^] to ensure that the transgenes are overexpressed throughout lifespan. The results showed that Tg(*TXN*)^+/0^ mice overexpress Trx1 in all tissues over their lifespan and that no compensatory down-regulation occurs in Trx2, glutathione, glutaredoxin, or other major antioxidant enzymes. Although Tg(*TXN*)^+/0^ mice showed similar levels of overexpression compared to the Tg(act-*TXN*)^+/0^ mice, we did not observe a significant increase in survival of Tg(*TXN*)^+/0^ mice, i.e., these mice showed a slightly increased survival in earlier lifespan, which was not significant, and appeared to have a higher mortality thereafter. These survival data are consistent with our previous study with Tg(act-*TXN*)^+/0^ mice, which showed that overexpression of Trx1 significantly increased the earlier part of lifespan in males and slightly increased the earlier lifespan in females but showed no extension of maximum lifespan in either males or females [20]. The cross-sectional pathological analysis of old mice (22–25 months old) indicates that the Tg(*TXN*)^+/0^ mice had a significantly higher severity of lymphoma and more tumor burden than WT mice. The accelerated tumor development in old mice in this study is also consistent in part with our previous study with Tg(act-*TXN*)^+/0^ mice, in which end-of-life pathology of Tg(act-*TXN*)^+/0^ mice had a slightly higher incidence of fatal lymphoma compared to WT mice [20].

These results suggest that increased levels of Trx1 over the lifespan in the Tg(*TXN*)^+/0^ mice showed some beneficial life-extending effects in the earlier part of lifespan, which were consistent with our previous observations, but continuous overexpression of Trx1 showed no significant effects on median or maximum lifespans. Therefore, Trx1 overexpression does not have beneficial effects in old animals. In fact, continuous overexpression of Trx1 possibly had deleterious effects in the later part of lifespan. This is supported by our observations that Tg(*TXN*)^+/0^ mice appeared to have a higher mortality in the later part of lifespan, had a significantly higher severity of lymphoma, and more tumor burden than WT mice at 22–25 months of age, which are also similar to previous observations with Tg(act-*TXN*)^+/0^ mice [20].

The increased cancer development due to Trx1 overexpression is not a surprising observation because Trx1 is overexpressed in various cancers [39], and Trx1 plays important roles in cancer development [39]. Although most studies that involve investigations of tumors suggest a possible role of Trx1 in carcinogenesis, our study is the first to demonstrate that Trx1 overexpression plays important roles to promote spontaneous tumor development with age *in vivo*.

Although Tg(*TXN*)^+/0^ mice showed accelerated cancer development, the later part of lifespan was similar to WT mice, i.e., lifespan was not shortened by enhanced tumor development. These observations were consistent with our previous studies that also demonstrated that the interventions (genetic, dietary, and others), which change the incidence/development of one disease (e.g., cancer), did not change the lifespan in rodents. For example: 1) MnSOD heterozygous KO mice had an increased incidence of tumors, however, these mice had a similar lifespan compared to WT mice [40]; 2) single housed calorie restricted (CR) male C57BL/6 mice had less fatal tumors, but the lifespan was similar to multiple housed CR mice [29]; and 3) CR with exercise reduced the fatal tumor incidence in F344 rats, but the maximum lifespan was similar to sedentary CR rats [41]. Therefore, changes in one disease does not always have a maximum impact on lifespan. Furthermore, in this study, we did not observe changes in the total number of diseases per mouse (disease burden), which could be a better index if the intervention could affect ‘aging’ more. Previously, we observed that extended lifespans of long-lived animals were accompanied by less disease burden than the control groups [30,31]. Therefore, little effects on disease burden by Trx1 overexpression could be one of the reasons increased cancer development alone did not have a significant impact on lifespan.

Our data show that continuous overexpression of Trx1 in mice reduced oxidative damage *in vivo*. Although oxidative damage has been shown to play important roles in carcinogenesis [40], our results show that reduced oxidative damage in the cells/tissues appeared to have little influence on spontaneous tumor formation in old mice. Furthermore, our results also show that reduced oxidative stress had little effects on lifespan. A series of comprehensive studies with transgenic mice overexpressing various antioxidant enzymes demonstrated that antioxidant overexpression protects cells/tissues from oxidative stress and shows little effects on lifespan [42]. Thus, our data along with others’ could indicate that reduced oxidative damage to cells/tissues alone may not be sufficient enough to have significant effects on lifespan.

The next question is why does Trx overexpression promote cancer development in old mice? Trx is well-known for its anti-apoptotic effects by the inhibition of the ASK1 pathway [14,20,43–45], and reduced apoptosis could be one of the contributing factors for cancer onset/progression [46]. Our current and previous studies also show that Trx1 overexpression in mice inhibits the ASK1 pathway, which could facilitate cancer growth in old animals. The inhibition of the ASK1 pathway by Trx1 overexpression did not change the mitochondrial apoptosis pathway. Therefore, the suppression of the ASK1 pathway may be promoting cancer development, which resulted in the accelerated mortality in old Tg(*TXN*)^+/0^ mice. Thus, the continuous overexpression of Trx1 may not only show lifespan extension but also promotes cancer development in older mice that may already harbor transformed cells.

Our findings suggest that the increased levels of Trx1 over the lifespan in mice showed a slight extension in the earlier part of lifespan but showed no life-extending effects in the later part of life, which was associated with enhanced tumor development in old mice. Enhanced tumor development was correlated to the suppression of the ASK1 pathway, which may be a contributing factor that promotes cancer development by Trx1 overexpression. The studies with two lines of Trx1 transgenic mice [20] and their interesting results have led us to two new questions: 1) does the overexpression of Trx in mitochondria (Trx2) attenuate aging and/or age-related pathology and 2) does the overexpression of Trx in both the cytosol (Trx1) and mitochondria (Trx2) show additive anti-aging effects? These studies will give us more information regarding the role of Trx in aging and age-related diseases, especially spontaneous cancer development, and their underlying mechanisms.
